# Benthic Habitat Mapping Using Multispectral High-Resolution Imagery: Evaluation of Shallow Water Atmospheric Correction Techniques

**DOI:** 10.3390/s17112639

**Published:** 2017-11-16

**Authors:** Francisco Eugenio, Javier Marcello, Javier Martin, Dionisio Rodríguez-Esparragón

**Affiliations:** Instituto Oceanografía y Cambio Global (IOCAG), Universidad de Las Palmas de Gran Canaria (ULPGC), Campus Universitario de Tafira, 35017 Las Palmas de G.C., Spain; javier.marcello@ulpgc.es (J.M.); javier.martin@ulpgc.es (J.M.); dionisio.rodriguez@ulpgc.es (D.R.-E.)

**Keywords:** high-resolution imagery, atmospheric models assessment, sunglint correction, coastal water ecosystem, benthic mapping, bathymetry retrieval

## Abstract

Remote multispectral data can provide valuable information for monitoring coastal water ecosystems. Specifically, high-resolution satellite-based imaging systems, as WorldView-2 (WV-2), can generate information at spatial scales needed to implement conservation actions for protected littoral zones. However, coastal water-leaving radiance arriving at the space-based sensor is often small as compared to reflected radiance. In this work, complex approaches, which usually use an accurate radiative transfer code to correct the atmospheric effects, such as FLAASH, ATCOR and 6S, have been implemented for high-resolution imagery. They have been assessed in real scenarios using field spectroradiometer data. In this context, the three approaches have achieved excellent results and a slightly superior performance of 6S model-based algorithm has been observed. Finally, for the mapping of benthic habitats in shallow-waters marine protected environments, a relevant application of the proposed atmospheric correction combined with an automatic deglinting procedure is presented. This approach is based on the integration of a linear mixing model of benthic classes within the radiative transfer model of the water. The complete methodology has been applied to selected ecosystems in the Canary Islands (Spain) but the obtained results allow the robust mapping of the spatial distribution and density of seagrass in coastal waters and the analysis of multitemporal variations related to the human activity and climate change in littoral zones.

## 1. Introduction

Coastal ecosystems are characterized by high biodiversity and primary production. Unfortunately, they are very sensitive to changes due to human activity, natural phenomenon, introduction of non-native species and other factors. So, given the importance of coastal water ecosystems for life quality and the global climate, systematic and efficient information is important for the monitoring of such protected areas. Consequently, the capability to map and monitor these benthic environments is fundamental for developing the corresponding management policies [1,2]. In this context, satellite remote sensing is a useful technology to monitor such coastal shallow ecosystems [3]. However, these areas are challenging as turbidity is usually present and, therefore, to derive parameters such as bathymetry or bottom features, the effects of the water column have to be accounted for. 

Estimating the water reflectivity using a radiative model has provided satisfactory results as it considers the water absorption and backscattering phenomena and the relationship between the seafloor albedo and the reflectivity of the shallow waters due to multiple scattering. Consequently, the recorded signal is an integration of contributions from the water column and from the bottom. In this line, some research has been performed and sophisticated models have been developed to properly separate individual signals [4,5,6]. Besides, to map bathymetry or benthic habitats, optical remote seafloor reflectance requires representations of the water-leaving reflectance spectrum as a function of depth for realistic bottom materials and water composition [7,8,9].

Recently, an increase in the availability of high spatial resolution satellite imagery has been possible thanks to the launch of new platforms, e.g., the WorldView series, providing a cost-effective solution to generate benthic maps [10,11]. Thus, advanced satellite image processing techniques offer a reliable, quantitative and cost-effective alternative for benthic mapping when compared to aerial photo-interpretation. Different benthic habitat mapping approaches based on satellite data have demonstrated to be effective [4]; however, results of high-resolution imagery-based benthos classification are affected, as indicated, by water column effects, bottom materials and, to a great extent, by the atmospheric absorption and scattering. Thus, atmospheric correction is an essential pre-processing of such remote sensing imagery and, accordingly, diverse techniques have been developed over the years [12,13]. 

After an extensive literature review of the state-of-the-art, they are commonly categorized as imaged-based methods as well as in more advanced physical-models based on the radiative transfer theory (RTM). In the first group, image-based techniques, a number of methods have been developed to remove or reduce atmospheric effects and retrieve the surface spectral reflectance. These methods vary from direct digital number transformation to reflectance [14], Dark Object Subtraction (DOS) of atmospheric path radiance [15,16], COsine eStimation of aTmospheric (COST) transmittance [17] to QUick Atmospheric Correction (QUAC) [18]. On the other hand, the most used advanced physical-based radiative transfer models are the Fast Line-of-sight Atmospheric Analysis of Spectral Hypercubes (FLAASH), the ATmospheric CORrection (ATCOR) and the Second Simulation of a Satellite Signal in the Solar Spectrum (6S), respectively [19,20,21]. Several authors have compared the performance of these methods for Landsat TM and ETM+, Quickbird, Hyperion and other remote sensing systems. 

A few comparative studies covering several methods have been carried out using medium and high-resolution data, for example Mahiny and Turner (2007) [13] carried out a study of four atmospheric correction methods (two relative approaches: pseudo-invariant features (PIF) and the radiometric control sets (RCS), COST and 6S models) on Landsat TM scenes; Nguyen et al., 2015 [16] analyzed 3 models (DOS, FLAASH and 6S) using Landsat ETM+ and, finally, in Marcello et al., 2016 [22] the performance of 5 representative atmospheric correction algorithms (DOS, QUAC, FLAASH, ATCOR and 6S) was assessed, using high-resolution WorldView-2 imagery. A brief summary is presented in Table 1. In general, model-based techniques work much better than image-based but not a single algorithm has demonstrated a superior performance in all the scenarios.

Although spectral reflectance retrieved from sophisticated RTM-based physical-models has often relatively high accuracy, RTM require in-situ measurements, parameters about the state and composition of the atmosphere at the time of satellite overflight, as spectral optical thickness of various atmospheric components, atmospheric and aerosol models, etc. For most potential users of high-resolution images in coastal monitoring and, specifically, WorldView-2 imagery, we found that these studies mainly covered some methods, typically applied to mapping vegetation and they did not assess, in detail, the influence of the atmosphere conditions in littoral zones water-leaving radiances. 

For example, in our previous research [22], to properly use the high-resolution data for mapping vegetation, the performance of five representative atmospheric correction algorithms (DOS, QUAC, FLAASH, ATCOR and 6S) were assessed, using WorldView-2 imagery and field spectroradiometer data, with the goal of identifying the most appropriate techniques. The study also included a detailed analysis of the parameterization influence of the aerosol model and its optical thickness, parameters to be properly adjusted. This strategy consisted of a relative evaluation, changing the input factors (parameters and models) in the configuration to identify how they affect in the estimation of the surface reflectivity. Specifically, different inputs were adjusted in the analysis: atmospheric model, aerosol model, aerosol optical thickness, adjacency effect and altitude. The effects of the atmospheric correction were studied in vegetation and soil sites belonging to different protected semi-arid ecosystems. As demonstrated in our previous research [22,23], to monitor natural protected areas, commonly used image-based atmospheric correction methods such as DOS, COST and QUAC don’t work properly for WorldView-2 high-resolution imagery.

Consequently, in the atmospheric modeling context, this work presents a detailed study of 3 physical model-based atmospheric correction algorithms (FLAASH, ATCOR and 6S) applied to the 8 bands of the high-resolution WorldView-2 satellite. The effects of the corrections have been studied in representative protected coastal ecosystems. This study also includes a detailed analysis of sea surface reflectance compared with in-situ spectral data collected, in real scenarios, at the time of satellite overflight. 

In addition, depending on the zones, WorldView-2 data acquired for coastal zones are often severely affected by glint, the light reflected on the crests or slopes of waves. Accordingly, estimations of water quality or the seafloor albedo are seriously impeded by the specular reflection of solar radiation on rough water surfaces (sunglint). This challenging topic could be addressed applying previous models and methods designed to take advantage of the glint to get surface information (e.g., wave height) or to eliminate glint contamination before estimating water-leaving reflectance [26,27]. 

In this work, following the method described in [28], an automatic deglinting procedure was implemented, integrated in the Radiative Transfer Modeling (RTM) inversion, to remove sea surface effects from high-resolution imagery in shallow-water environments, prior to estimating seafloor reflectivity, which allows computing the water Inherent Optical Properties (IOPs), bathymetry and seafloor albedo contributions.

Finally, a relevant application of the proposed atmospheric correction combined with the automatic deglinting procedure for benthic habitats mapping in shallow-waters marine protected environments is presented. The coastal bottom mapping is possible due to the integration of a linear mixing model of benthic classes within the radiative transfer model of the water, which allows us to obtain the abundances of the benthic classes under study. It is important to emphasize the difficulty to obtain accurate benthic maps as complex models are required and favorable sea state conditions and satellite geometry are necessary, as studied in [29]. Due to the great perturbations (attenuation and back-scattering) caused by the atmosphere, with respect to the low amount of energy provided by the aquatic environment, the joint modeling of these two elements has been required to eliminate the atmospheric component of the reflectivity obtained by the satellite. Similarly, the radiative model of sea water allows the identification of the backscatter and attenuation components, enabling, for example, the modeling of the reflectivity component due to the water and due to the coastal seafloor. The main application of the seafloor albedo is the detection and classification of the different types of benthic habitats found on the seabed, allowing the robust mapping of the spatial distribution and density of seagrass in coastal waters and the analysis of multitemporal variations related to the human activity and climate change in littoral zones. 

In summary, a complete strategy has been developed for the processing of high-spatial-resolution WorldView-2 imagery. Three physical model-based atmospheric correction algorithms (FLAASH, ATCOR and 6S) have been analyzed, implemented and validated over a database of in-situ measurements collected during field campaigns, simultaneously to the satellite overflight, in order to select the method achieving the best performance. The different types of seafloor covers have been modeled applying linear unmixing of pure spectral signatures of the main benthic classes. Accurate bathymetric mapping was also obtained as this information is very important in benthic habitats modeling. It is important to emphasize that most research published, about the mapping of benthic habitats, deals with ideal scenarios where coral reefs are monitored in very calm, clear and shallow waters. In this work, though, more complex ecosystems are considered.

## 2. Methodology

### 2.1. Dataset and Study Area 

The launch of the WorldView-2 satellite, at the end of 2009, marked a new milestone in the state of the art of very high-resolution satellites, providing a spatial resolution of 0.46 m in the panchromatic (PAN) band and 1.84 m in eight multispectral (MS) channels, with additional bands (coastal, yellow, red edge and a second near-infrared) not included in previous high-resolution platforms (i.e., Ikonos, Quickbird, Geoeye, Quickbird, etc.). WV-2 introduces an unusually high number of channels for this type of satellites, including a high penetration blue band that allows improving the capabilities of this satellite in the monitoring of coastal waters. In this line, DigitalGlobe [30] has decided to continue these services by launching WorldView-3 and Worlview-4 satellites in August 2014 and November 2016, respectively. Both platforms maintain the same number of bands in the optical-NIR range improving the spatial resolution (0.31 m PAN and 1.24 m in the MS channels) and adding new bands in the NIR (DigitalGlobe, Inc. , Westminster, CO, USA) (2017)). This study is based on level-2 Ortho Ready Standard and Radiometric-Corrected WorldView-2 imagery. 

**Figure 1:**
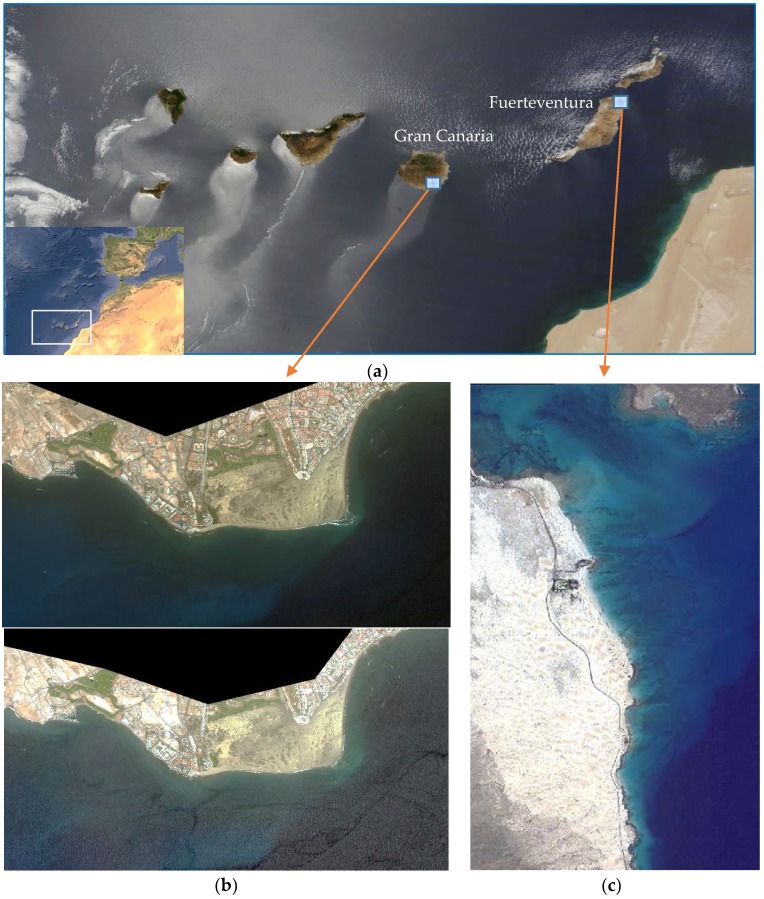
(**a**) Location of study areas (Canary Islands) (NASA’s Earth Observatory best satellite image of Earth 2013© (Canary Islands off the northwest coast of Africa, captured by NASA’s Terra satellite June 2013)) and; (**b**,**c**) WorlView-2 images of the two Canary Islands singular littoral zones: (**b**) Maspalomas area (Gran Canaria Island), **upper**: 11 August 2013; **lower**: 4 June 2015 and; (**c**) Corralejo-Lobo Island area (Fuerteventura Island) 28 October 2010.

The study area selected is the Canary Islands (Spain), off the northwest African coast. Particularly, shallow coastal ecosystems with clear waters and easy access for the collection of field data, as shown in Figure 1. Specifically, the littoral protected zones selected are: Maspalomas, in the south part of Gran Canaria Island and Corralejo-Lobos Island (Fuerteventura Island), as shown in Figure 1b,c, respectively. 

The Natural Reserve of the Dunes of Maspalomas, which covers an area of approximately 403.9 hectares, consists of three ecosystems: palmeral, inner-lake and the dunes. This natural resource extends into the sea creating a perfect habitat for the seagrass beds, which have a great importance in the biodiversity of the Canary Islands coast. For the coast of Corralejo-Lobos Island, monitoring of water quality is of special interest, in addition to being a UNESCO Biosphere Reserve, due to its proximity to the inter-island channel that separates Fuerteventura and Lobos Islands, where, in turn, important water currents are generated and, consequently, oceanographic structures at local scale can be observed using the diffuse attenuation coefficient of the water *kd (490)* as a tracer mode [31]. 

Detailed information on Canary Islands WV-2 imagery and the validation data used are shown in Table 2. The multispectral images considered in our analysis were acquired by the WV-2 satellite on 11 August 2013, 4 June 2015 (both of Gran Canaria Island) and 28 October 2010 (Fuerteventura Island). The incident angles (mean off-nadir viewing angle) of the upper and low left images, in Figure 1, are 9.5° and 7.9°, respectively. The Corralejo-Lobos image (incident angle 8.7°) is almost sunglint free and seafloor is clear, as shown in Figure 1c, while the Maspalomas imagery are severely contaminated by sunglint due to sea surface waves and so the bottom texture is invisible in both images (see Figure 1b).

**Figure 2:**
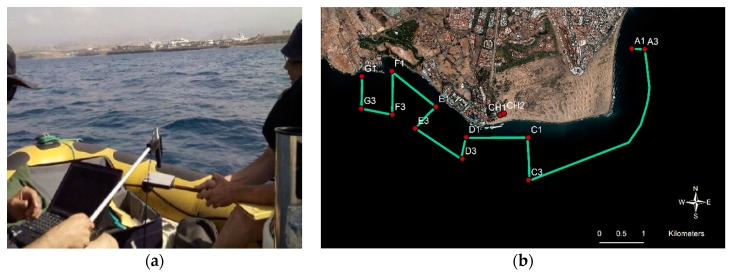
(**a**) Procedure of ground-based spectral data acquisition with the ADS Fieldspec 3 and; (**b**) ship transects and sampling sites during the Maspalomas field campaign of June 2015.

As indicated, in order to validate the satellite reflectance obtained in the surface, after the atmospheric correction, a field spectroradiometer was used for an in-situ sampling. Specifically, the ADS Fieldspec 3 instrument (see Figure 2a) was recording the in-situ reflectance above the sea water in the optical and NIR bands nearly coincident with the WorldView-2 satellite over flight in two seasons: August 2013 and June 2015.

Likewise, the water quality of the Maspalomas shallow coastal ecosystem has been sampled in order to obtain chlorophyll-a, turbidity and CDOM concentrations and to collect seafloor information, specifically bathymetry and benthic habitats, a digital echosounder, video records and a differential GPS receiver were used. Figure 2b shows the geographic location of the in-situ transects and sampling sites, during the Maspalomas oceanographic campaign of 4 June 2015 (similar locations were sampled during 11 August 2013).

### 2.2. Multispectral High-Resolution Data Correction 

As mentioned, the low water reflectivity implies small radiation levels reaching the satellite sensor from water regions. Consequently, radiometric calibration and atmospheric correction are extremely important for the robust monitoring of coastal zones using satellite data. In these situations, the atmosphere provides the major contribution to the sensed signal, which is also affected by the sensor-target-illumination geometry and the radiometric calibration.

Therefore, there are two different steps that must be addressed: sensor calibration and correction of atmospheric effects. As first stage, the acquired WV-2 images are radiometrically calibrated to obtain the radiance physical values from the image digital values. ToA (Top of Atmosphere) radiance is defined as the energy reflected by the Earth surface and the vertical column of the atmosphere entering through the sensor at the height of the satellite, 770 km for the WV-2. The conversion from satellite data, radiometrically corrected, to radiance values is performed by,

(1) $$L_{\lambda }^{sen}=\frac{K_{Band}\;\cdot q_{Pixel,Band}}{\Delta \lambda_{Band}}$$

where $L_{\lambda }^{sen}$ represents the radiance value of the sensed band, $K_{Band}$ is the radiometric calibration factor of each band, $q_{Pixel,Band}$ is the radiometrically corrected image and $\Delta \lambda_{Band}$ is the effective bandwidth for each specific band.

These data are then converted to surface reflectivity, where the radiance values are normalized according to the illumination conditions and the absorption and back-scattering effects of the atmosphere are corrected, allowing to compute the spectral diffuse reflectance of the surface (assuming a Lambertian surface), by means of [32]:

(2) $$\rho_{su,\lambda }=\frac{\left(L_{\lambda }^{sen}-L_{a,\lambda }\right)\cdot d_{ES}^{2}\cdot \pi }{\tau_{v\lambda }\left(E_{o,\lambda }\cdot \cos\theta_{s}\cdot \tau_{s\lambda }+E_{d,\lambda }\right)}$$

**Figure 3:**
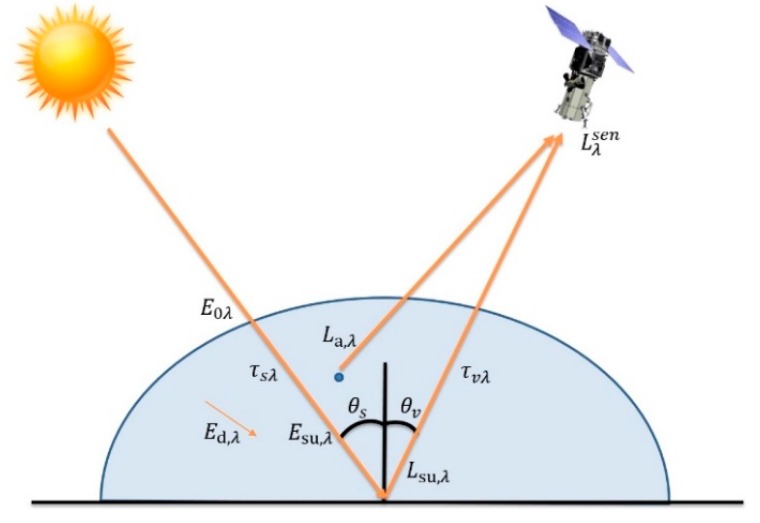
Model of atmospheric influence.

As it can be seen in Figure 3, $L_{\lambda }^{sen}$ is the radiance received by the satellite,$\;L_{a,\lambda }$ is the radiance contribution by the atmospheric dispersion for the band of wavelength λ, $d_{ES}^{\;}$ is the distance between the Earth and the Sun, $\tau_{v\lambda }$ is the atmospheric transmissivity for the upward flow, $E_{0,\lambda }$ is the solar irradiance at the top of the atmosphere, $\theta_{s}$ is the angle of the incident flux formed between the vertical and the solar rays, $\tau_{s\lambda }$ is the atmospheric transmissivity that the solar radiation crosses in the downward direction and $E_{d,\lambda }$ is the diffuse irradiance, as a consequence of the scattering and that depends on the conditions of the atmosphere.

#### 2.2.1 Atmospheric Correction Algorithms 

Coastal waters contain constituents other than phytoplankton such as suspended sediments and dissolved organic matter. Such additional components make these waters optically more complex than clear areas in deeper waters. Traditionally, atmospheric correction methods have been developed for retrieving water-leaving radiances over ocean waters and low resolution sensors (NASA-MODIS, ESA-SENTINEL3, etc.). However, in coastal shallow environments, the water-leaving radiance may be significantly higher due to the influence of the bottom albedo and the suspended and dissolved material back-scattering. Accordingly, applying deep ocean algorithms to satellite imagery acquired over such turbid coastal waters often result in erroneous retrievals [33,34] and, in consequence advanced atmospheric correction models are necessary for coastal waters.

As analyzed and explained in the Introduction section (i.e., see Table 1), different strategies have been developed and, in this work, the following three advanced physical-models based on the radiative transfer theory (RTM) algorithms have been selected for the analysis: FLAASH, ATCOR and 6S.

The atmospheric correction model FLAASH [8] derives its physics-based algorithm from the MODTRAN-4 radiative transfer code (RTC). FLAASH removes the scattering and absorption atmospheric effects to obtain reflectance at the surface by [22]:

(3) $$L_{TOA}=\left(\frac{A\rho_{SU}}{1-\rho_{e}S}\right)+\left(\frac{B\rho_{e}}{1-\rho_{e}S}\right)+L_{o}$$

where $\rho_{SU}$ is the surface reflectance, $\rho_{e}$ is the mean surface reflectance for the pixel and the surrounding region, ***S*** is the spherical albedo of the atmosphere, $L_{o}$ is the radiance that atmosphere backscatters and coefficients ***A*** and ***B*** depend on the atmospheric and geometric conditions. The first term in Equation (3) relates to the direct radiance from the surface to the sensor, while the second one corresponds to the radiance from the surface that is scattered by the atmosphere into the sensor (see Figure 3). The difference between $\rho_{SU}$ and $\rho_{e}$ accounts for the spatial mixing of radiance among nearby pixels (adjacency effect) caused by atmospheric scattering. A, B, S and $L_{o}$ can be empirically determined from the MODTRAN-4 simulations for a specified atmosphere model (in our case, maritime model). The viewing and solar angles of the measurement and nominal values for the surface elevation, aerosol type and visible range for the scene must be specified. 

For the ATCOR [35] algorithm, also based on MODTRAN-4 RTC, the surface reflectance, without taking into account the adjacency effect, is obtained by [22]:

(4) $$\rho_{SU}=\frac{1}{a_{1}}\left(\frac{d^{2}\pi L_{TOA}}{E_{TOA}\cos\theta_{i}}-a_{0}\right)$$

where $a_{0}$ and $a_{1}$ coefficients are obtained from the estimation of the main atmospheric parameters: water vapor column, aerosol type and optical thickness. In this work, for the atmospheric pre-processing of WorldView-2 data with the ATCOR algorithm, a two-step procedure has been considered: the first involved getting the atmospheric effect assuming an isotropic or Lambertian reflectance law applying Equation (4); while the second step corrected the adjacency effect [22].

The 6S [36,37] is an advanced radiative transfer code designed to simulate the reflection of solar radiation by a coupled atmosphere-surface system for a wide range of atmospheric, spectral and geometrical conditions. This algorithm predicts the top of atmosphere (TOA) reflectance $\rho_{TOA}$ using information about the atmospheric conditions and surface reflectance. 

6S defines $\rho_{su}$ as the surface reflectivity of the cover, surrounded by a homogeneous environment of reflectivity $\rho_{e}$, while the ToA reflectivity is defined as $\rho_{TOA}$, which is defined as follows [32]:

(5) ρTOA(θs,θv,Δϕ)=tg(θs,θv){ρa(θs,θv,Δϕ)+[e−τμs+td(θs)]ρsue−τμv+ρetd(θv)1− ρeS}

Reference to the wavelength has been removed for better clarity of the equation. The meaning of each term is next detailed:

- $\mu_{s}=\cos\left(\theta_{s}\right),\;\mu_{v}=\cos\left(\theta_{v}\right).$
- Δϕ represents the difference between solar and satellite azimuth.
- $t_{g}$ represents the total transmissibility of the gases (in the upward and downward path), taking into account the absorption of the different gases of the atmosphere.
- $\rho_{a}$ represents the atmospheric reflectivity, which depends on the molecular properties and the aerosols in the atmosphere.
- τ represents the atmospheric thickness (Atmospheric Optical Depth, AOD).
- $t_{d}\left(\theta_{s}\right),\;t_{d}\left(\theta_{v}\right)$ represents the diffuse transmittance of the atmosphere.
- S represents the spherical albedo of the atmosphere.
- The $\left(1-\rho_{e}S\right)$ term takes into account the multiple scatterings between the surface and the atmosphere. As it can be seen, the absorption and scattering processes are dealt separately in the 6S equation. The scattering produced by molecules and aerosols are differentiated as well. The total reflectivity of the atmosphere is obtained by the introduction of coefficients from Rayleigh scattering and aerosols. These coefficients are obtained by first-order approximations.

6S is a single layer model, where the variations of the parameters in the vertical column of the atmosphere are not taken into account. Equation (5) is a monochromatic expression. To obtain the result of a single multispectral band, 6S computes the equation for the entire range of wavelengths with a step of 5 nm, integrating all these results to get the final result for the specific band according to the spectral response of the sensor for such wavelength. 

The input parameters requited by 6S are the type of sensor, date and time of image acquisition, geographical coordinates of the scene center, visibility (aerosol optical thickness) and the sun zenith and azimuth angles. 

In this study, a strategy based on the absolute evaluation has been applied to compare the atmospheric correction methods. This approach compares the reflectivity of the atmospherically corrected image pixels with the field measurements recorded using the ADS Fieldspec 3 spectroradiometer. Really, to accurately compare both datasets, the reflectivity measured by the spectroradiometer in very narrow channels was pre-processed to properly accommodate the WV-2 spectral bands. Representative coastal shallow water (points A to G) and inner-lake water (points CH) sites included in the analysis are shown in Figure 2 and Table 3 (Maspalomas field campaign, 4 June 2015). 

Special care was taken into account to generate the match-ups between field and satellite locations in order to guarantee that we are comparing the same sites. However, precise references are not available in water areas and, in consequence, a 3 × 3 window around the GPS location was considered to account for the existing satellite and ground geometric errors. 

We applied two error analysis criteria, namely, the Root-Mean-Square Error (RMSE) which was computed to quantify the estimated reflectivity differences and the BIAS which was used to determine the tendency to overestimate or underestimate as regards field data. The equations used to calculate the error criteria are as follows:

(6) $$\;RMSE=\sqrt{\sum_{i=1}^{N}\frac{{\left(\rho_{in-situ}{}_{i}-\rho_{sat}{}_{i}\right)}^{2}}{N}};\;BIAS=\sum_{i=1}^{N}\frac{\left(\rho_{in-situ}{}_{i}-\rho_{sat}{}_{i}\right)}{N}\;$$

where $\rho_{in-situ}$ is the measured in-situ surface reflectance and $\rho_{sat}$ is the surface reflectance estimated from the satellite data. The closer to 0 is the RMSE the better the algorithm that models the reflectivity, implying a smaller difference between the in-situ reflectance values and those estimated from the image. Negative BIAS implies that the algorithm tends to overestimate the real observation.

In many applications, the appropriate setting is important for an accurate atmospheric correction. In our case, the analysis of the different input parameters in the estimated reflectance led to slight changes as a consequence of the selection of the appropriate inputs for each area and date. The effects of parameter settings of the different physical models (FLAASH, ATCOR and 6S) are for both areas of study [22,23]:

(i) The Mid-Latitude Summer seems the most suitable atmosphere model for the climate of the Canary Islands. Water vapor was considered implicitly when selecting the atmosphere model as it considers standard column water vapor amounts (from sea level to space).
(ii) The most acceptable aerosol model for the islands is the Maritime model.
(iii) The aerosol optical thickness (AOT) parameter must be properly adjusted using in-situ or satellite information because major errors in their estimation can significantly affect the surface reflectivity computed. Nowadays, such information is daily available from satellite sensors (i.e., MODIS at 550 nm).

#### 2.2.2. Automatic Correction of Sunglint Effect

As previously indicated, the removal of sunglint is necessary for the reliable retrieval of bathymetry and seafloor mapping in shallow-water environments. In this context, sunglint correction methods have been developed for open ocean imaging and high-resolution coastal applications as reviewed in [26]. However, algorithms using the NIR channel to eliminate sunglint [38] are not appropriate in coastal areas because, due to the turbidity and seabed albedo, the water reflectivity in the NIR band is not always negligible. 

**Figure 4:**
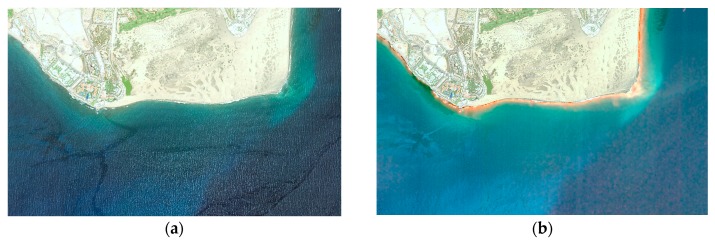
(**a**) WorldView-2 color composite image of Maspalomas (Gran Canaria Island, June 2015) and (**b**) image after sunglint correction using the algorithm implemented in [28].

To overcome this problem, Martin et al., 2016 [28] proposed to integrate the sunglint removal algorithm in the radiative transfer model to estimate the contribution of the NIR reflectance of coastal waters, which will allow us to eliminate this contribution of the specular NIR reflectance. As an example, Figure 4 shows a color composite image before and after the sunglint contamination removal. After sunglint elimination, bright spots distributed along the wave edge basically disappear and the seafloor becomes much clearer (i.e., seagrass patches in the upper right side of the image). In this example of combined atmospheric-deglinting methodology, the atmospheric correction of the eight WV-2 bands using the 6S model has been applied [11]. 

Next, in this work, using the WV-2 channels completely corrected, after the removal of atmospheric and sunglint effect, the radiative transfer equations (RTE) are calculated. From this calculation the intrinsic properties of the substances contained in the water (IOPs: Inherent Optical Properties) are determined, the depth of the water column is retrieved (bathymetry) and the albedo of the coastal bottom is estimated. Finally, using the reflectivity information from the channels of greater penetration and the bathymetric information, it is possible to obtain maps of the seafloor albedo and the abundances of pure classes modeled in the linear unmixing. In this context, abundance maps for each class indicate the factional coverage of each type of seafloor in each pixel. Thus, these maps indicate the percentage of coverage of each class in a mixed pixel and, in consequence, pure pixels have a value of 1, specifying that the 100% of the pixel is composed by a single pure class. 

### 2.3. Coastal Monitoring Algorithms: Benthic Habitat Abundance Mapping and Bathymetry Estimation

As analyzed in our recent research published in Eugenio et al., 2015 [11]: “In Canary Islands, as well as other parts of the world, coastal seafloor benthic habitats (and seagrass density) have traditionally been mapped from aerial photography, using photointerpretation techniques, or using in-situ measurements (bionomic maps obtained with oceanographic ships).” In that work, spatial and spectral image processing techniques were combined to map benthos types, extent and density using WorldView-2 satellite imagery of Canary Islands. Particularly, the availability of spectral bands at short wavelength allowed the development of algorithms to study the seafloor and benthic vegetation, of high ecological importance and good bio-indicators of the quality of coastal waters. 

In our former research, a benthic habitat map was obtained combining the water column correction, seabed normalized indexes (seagrass, sand and rocks) and supervised classification algorithms. Support Vector Machine (SVM) [39], a machine learning classification method, was applied to the WorldView-2 corrected bands and benthic indexes. Training regions for each seabed class were defined and the Jeffries-Matusita distance was used to measure their spectral separability. Finally, the confusion matrix and the kappa coefficient were used to measure the maps quality.

The benthic mapping process is very challenging as depth and the dynamic inherent optical properties of light scattering and absorption of the water column change the spectral response of seafloor features over space and time [8,9,10,11]. With appropriate bathymetric information, water column light attenuation correction coefficients can be calculated for each band by comparing pixels of known bottom types at different depths as a depth variant light attenuation correction. 

The inversion of the marine optical model, to obtain a simultaneous and optimal solution of the optical parameters involved in the coastal water reflectance, provides the most suitable tool for the study of environments as dynamic as coastal waters. This approach, initially used in hyperspectral sensors, has been adapted to multispectral sensors with a sufficient number of bands, such as the WorldView-2 satellite. The modeling of the coastal bottom in shallow waters turns out to be of great importance in the correct computation of the water properties and the bathymetry, providing in itself interesting information to know the distribution of the different coastal benthic classes. 

**Figure 5:**
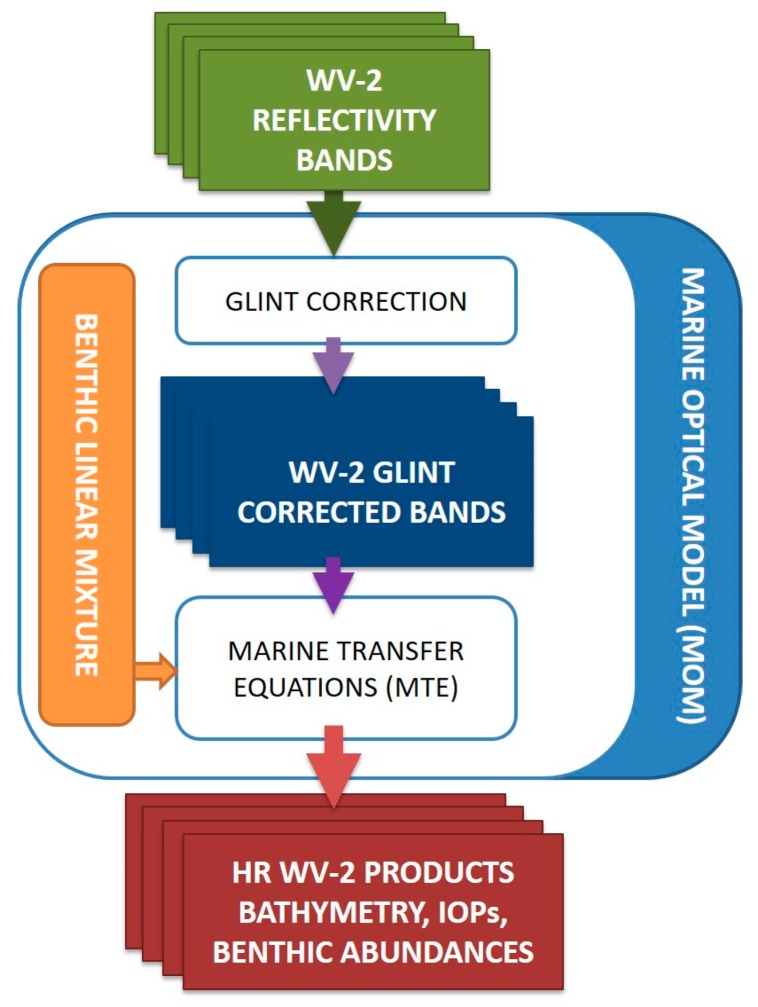
Proposed diagram of the radiative transfer model using linear mixture of benthic elements.

The following diagram, Figure 5, shows the workflow implemented in this work, where the different modules present in the model can be appreciated.

The linear mixing model for pure benthic species has been used for modeling the coastal bottom, where the reflectance is due to a linearly weighted mixture of pure elements according to their abundance. In this way, thanks to the integration of the linear mixing of pure benthic elements in the marine model, it is possible to obtain abundances maps of the seafloor, as well as obtaining the RGB image of the bottom, even of channels not detectable due to their poor penetration, like the red band, thanks to the knowledge of the spectral signatures of each modeled benthic element.

For the implementation of the radiative transfer model, based on the multispectral adaptation of the Hyperspectral Optimization Process Exemplar Model (HOPES) [40], the equation proposed by [41] is used, where the modeled reflectivity $r_{rs}^{m}$ is due to the inherent reflectivity of water $r_{rs,\infty }$ and to the reflectivity of the coastal seafloor $\rho_{alb}$. The Beer-Lambert law is used to weigh these contributions [42]:

(7) $$r_{rs}^{m}\left(\lambda \right)\approx r_{rs,\infty }\left(\lambda \right)\left(1-e^{-\left[\frac{1}{\mu_{s}^{sw}}+\frac{D_{u}^{c}}{\mu_{v}^{sw}}\right]k_{d}z}\right)+\frac{\rho_{alb}\left(\lambda \right)}{\pi }e^{-\left[\frac{1}{\mu_{s}^{sw}}+\frac{D_{u}^{b}}{\mu_{v}^{sw}}\right]k_{d}z}\;$$

where $\mu_{s}^{sw}$ and $\mu_{v}^{sw}$ correspond to the geometric component of the sun illumination and satellite viewing geometry in the aquatic environment, $k_{d}$ is the diffuse attenuation coefficient of water, which, like $r_{rs,\infty }$, depends on the IOPs. z is the depth of the coastal bottom. $D_{u}^{c}$ and $D_{u}^{b}$ are light diffusion factors for the two ascending sources: the vertical water column ($D_{u}^{c}$) and the bottom reflectivity ($D_{u}^{b}$). For the calculation of the inherent water reflectivity $r_{rs,\infty }$ the equation proposed by Lee et al., 2002 [43] has been used.

In order to obtain the biophysical water properties, the following parameters have been used: *G* corresponding to the dissolved matter attenuation coefficient at 440 nm [44]; *P* the chlorophyll attenuation coefficient at 440 nm [41] and *X* the suspended matter backscattering coefficient at 400 nm. In this way, the inherent reflectivity of the sea water can be obtained by the following expression [45]:

(8) $$r_{rs,\infty }=\;0.0512u\left(1+4.6659u-7.8387u^{2}+5.4571u^{3}\right)\times \left(1+\frac{0.1098}{\mu_{s}^{sw}}\right)\times \left(1+\frac{0.4021}{\mu_{v}^{sw}}\right)\;$$

where we can appreciate that there is a relationship of the inherent reflectivity with the cosines of the solar illumination and satellite vision angles ($\mu_{s}^{sw}$,$\mu_{v}^{sw}$) and where u is the Gordon parameter [46] that relates the backscattering and attenuation, which depend on the *G*-*P*-*X* parameters, as follows:

(9) $$u=\;\frac{b_{b}}{a+b_{b}}$$

In a similar way, the diffuse attenuation is defined as the sum of the water attenuation and backscattering [7]:

(10) $$k_{d}=\frac{a+b_{b}}{\mu_{s}}$$

Sea water attenuation is obtained by the following expression:

(11) $$a\left(\lambda \right)=a_{w}\left(\lambda \right)+a_{ph}\left(\lambda \right)+a_{dg}\left(\lambda \right)$$

where $a_{w}\left(\lambda \right)$ corresponds to the pure marine water attenuation, $a_{ph}\left(\lambda \right)$ is the chlorophyll attenuation (function of *P*) and $a_{dg}\left(\lambda \right)$ corresponds to the dissolved matter attenuation (function of *G*).

In turn, backscattering is obtained by the following expression:

(12) $$b_{b}\left(\lambda \right)=b_{bw}\left(\lambda \right)+b_{bp}\left(\lambda \right)$$

where $b_{bw}\left(\lambda \right)$ corresponds to the backscattering of the pure water and $b_{bp}\left(\lambda \right)$ to the backscattering of the suspended matter.

The phytoplankton attenuation is modeled as follows [47]:

(13) $$a_{ph}\left(\lambda \right)=(a_{0}\left(\lambda \right)+a_{1}\left(\lambda \right)\ln\left(P\right))\times P$$

where $a_{0}\left(\lambda \right)$ and $a_{1}\left(\lambda \right)$ are the two wavelength-dependent parameters, while P is the absorption value of the phytoplankton at 440 nm ($a_{ph}\left(440\right)$).

The disolved matter attenuation is modeled as follows [44]:

(14) $$a_{dg}\left(\lambda \right)=\;G\times e^{-S_{g}\left(\lambda -\lambda_{0}\right)}\;$$

where the $S_{g}$ parameter describes the degree of decay of the exponential function according to the wavelength and may have values ranging from 0.01 to 0.03 nm^−1^. The G parameter indicates the attenuation magnitude at a wavelength of 440 nm.

It can be observed how parameters modeled in the IOPs, as well as bathymetry, do not depend on the wavelength, while the bottom albedo does. For this reason, it is necessary to model it to obtain a good result in the inversion of the model. For this, Lee et al., 1999 [48] proposed a sandy bottom reflectivity model, which can be improved by using the bottom linear model FCLU (Fully Constrained Linear Unmixing) [49], as shown in the following equation:

(15) $$R\left(\lambda \right)=\overset{P}{\underset{i}{\sum }}ab_{i}\times em_{i}\left(\lambda \right)\;$$

where the modeled reflectivity is linearly proportional to the endmember abundance and its reflectivity. In this way, knowing the reflectivity of the pure benthic elements to be modeled, only the endmember abundances have to be modeled, thus being independent of the wavelength.

**Figure 6:**
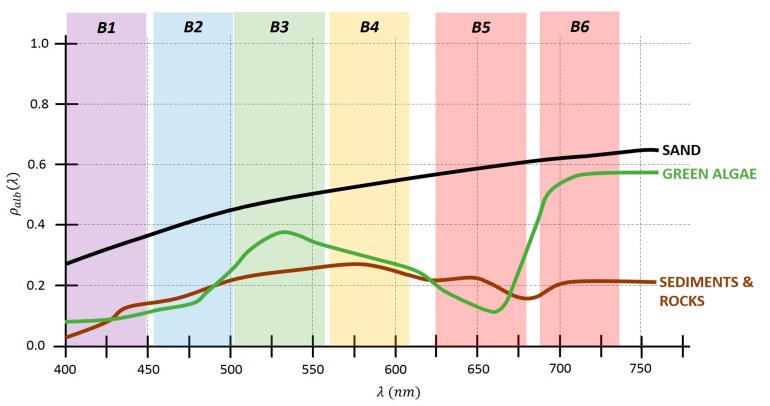
Normalized reflectivity of common pure classes in coastal seabed [50].

Due to the low contribution of the coastal bottom to the reflectivity of the WV-2 channels and due to the limitations of the multispectral bands, the most common benthic elements and with the most separable reflectivities have been used to allow the correct linear modeling. The first endmember selected has been sand, with high reflectivity and very common in coastal seafloor studies; sediments or low reflectivity rocks have been the second and, finally, algae has been the last one selected (seagrass and algae spectral signatures are very similar, thus a single endmember has been selected. The most common species in the Canarian seabed are *Cymodocea nodosa* and *Caulerpa prolifera*), where an average value of the most common varieties in the study area has been used. Figure 6 includes the spectral signature of the three endmembers used in the modeling with respect to the first six WV-2 bands.

Next, in the following system of equations the proposed radiative transfer model is shown, where the reflectivity of each of modeled WV-2 bands depends on the IOPs (*G*, *P*, *X*), on the bathymetry (*z*) and on the abundances of the pure benthic elements modeled. Note how the reflectivity of each band is obtained as the integration of the model results for this specific multispectral band according to the response filters of the WV-2 bands. 

Thus, using three pure elements, the total number of unknowns is 7 in a system of 8 equations, where a ninth one is added taking into account that the total sum of abundances of the endmembers must be the unit (FCLU).

(16) $$\;R_{rs}^{m}\left(band_{i}\right)=\underset{\lambda_{i}}{\sum }F\left(a_{w}\left(\lambda_{i}\right),b_{bw}\left(\lambda_{i}\right),a_{ph}\left(\lambda_{i}\right),\;a_{dg}\left(\lambda_{i}\right),\;b_{bp}\left(\lambda_{i}\right),\;z,\rho_{alb}\left(\lambda_{i}\right)\;\right)=\;F\left(G,P,\;X,\;z,\;ab_{EMs}\right)$$

where *i* refers to the WV-2 spectral bands (*i =* 1–6). Thus, in the radiative transfer modeling, this system of equations is inverted to obtain the parameters explained above. In order to achieve it, spectral minimization is used, whereby least squares minimize the error between the reflectivity of the WV-2 channels and the reflectivities obtained by the model as follows:

(17) $$\delta_{R_{rs}}=\frac{\sqrt{n}*\sqrt{\sum_{\lambda =1}^{n}{\left|R_{rs}^{m}\left(\lambda \right)-R_{rs}\left(\lambda \right)\right|}^{2}}}{\sum_{\lambda =1}^{n}R_{rs}\left(\lambda \right)}\;$$

where $\delta_{R_{rs}}$ is the error function to be minimized. To perform this minimization, the Levenberg-Marquardt [51] algorithm has been used for the iterative resolution of non-linear equation systems. Considering that we deal with an ill-posed problem, with multiple regional minimums and where each of the equations, which represent the multispectral bands, has a different level of water penetration. This fact implies that as depth increases, the quality of the coastal albedo results decrease. Nonetheless, this does not mean that the other parameters cannot be calculated, since it is a minimization procedure, not a resolution of a system of equations per se. The parameters that generate enough gradient in the cost function are optimized, while the parameters that do not affect are ignored.

The appropriate initialization of the variables to be optimized is very important in iterative algorithms. In our case, the depth z is fundamental since it is responsible of adjusting the component of reflectivity due to the water or the seabed albedo. Therefore, a heuristic algorithm has been used to initialize z. This algorithm, known as the ratio algorithm, uses the green and blue satellite bands and it has been adjusted for the albedo of coastal seafloors by using a high-resolution echosounder bathymetryas, as in [11]. 

## 3. Results and Discussion

### 3.1. Atmosperic Assessment: Absolute Evaluation Using In-situ Spectroradiometer Measurements 

In this section, a summary of the most relevant results achieved, in the atmospheric modeling context, for Canary Island coastal waters, using the WorldView-2 imagery are presented. The effects of the corrections have been studied in the Maspalomas protected coastal ecosystem (geographic locations of in-situ spectral data used in this work are provided in Table 4 and Figure 2). 

The overall results from the average measurements for the considered coastal shallow water and inner-lake points are included in Table 4, in which the RMSE and BIAS between the WorldView-2 corrected reflectance and in-situ measurement are presented.

**Figure 7:**
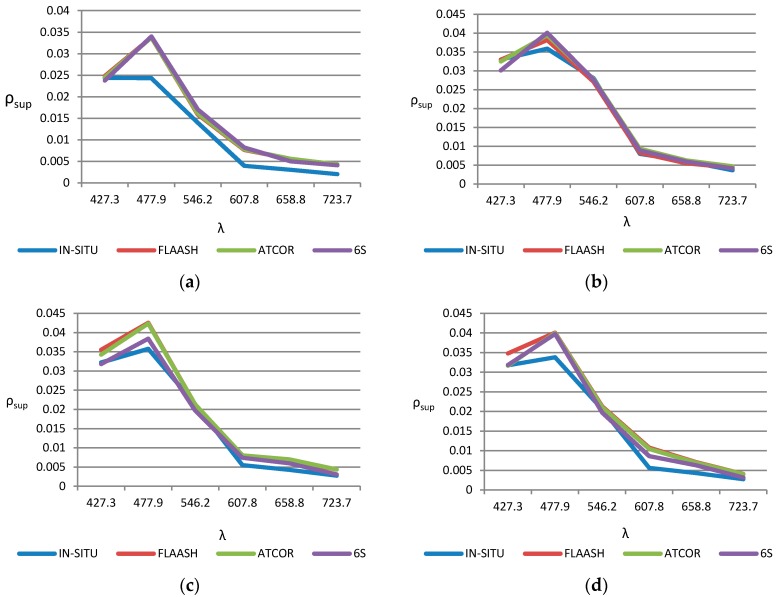

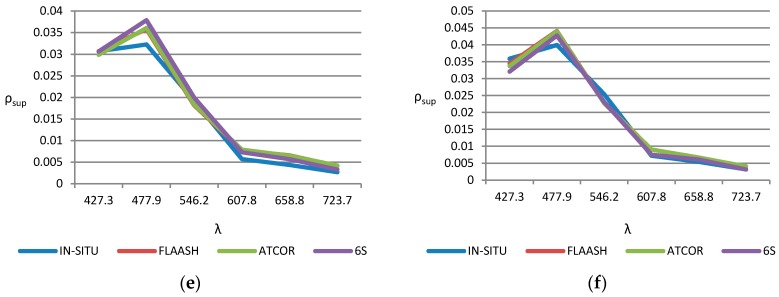
Spectral reflectivity signatures in the nearest coastal shallow water sites (points number 1 from A to G), as shown in Figure 2: (**a**) Coast point 1; (**b**) Coast point 4; (**c**) Coast point 5; (**d**) Coast point 8; (**e**) Coast point 10 and; (**f**) Coast point 12.

Results achieved by the three model-based atmospheric correction algorithms, FLAASH (red), ATCOR (green) and 6S (violet) are plotted in Figure 7, showing the detailed spectral reflectivity signatures, in selected locations, the nearest water points to the shore (see Figure 2), as well as the reference signature measured with the spectroradiometer (blue). 

The final result of the comparison between model-based algorithms provides, for coastal shallow waters, a better suitability of the 6S algorithm, according to the RMSE and BIAS values included in Table 4. This conclusion can be qualitatively observed in Figure 7, for the nearest points to Maspalomas littoral zone and WV-2 visible bands, where the differences between the corrected reflectance respect to in-situ reflectance, for the 3 atmospheric models, are relatively low. Specifically, the 6S model has the largest spectral correlation with ground-based measurements. The largest impact of the atmospheric reflectance corrections can be appreciated in the blue band (around 0.47 µm) and, in general, in visible spectrum, between 0.4 to 0.6 µm. On the other hand, ATCOR tends to overestimate the reflectance in bands above the green one, while FLAASH causes a general overestimate in all wavelengths.

**Figure 8:**
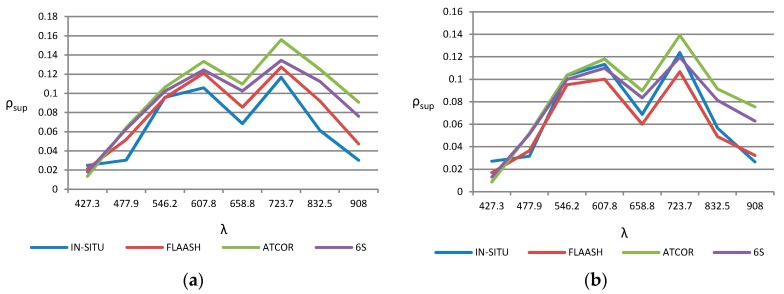
Spectral reflectivity signatures: (**a**) Inner-lake point CH-1 and; (**b**) Inner-lake point CH-2.

On the other hand, Figure 8 shows the sea surface reflectance, for all WV-2 bands (see Table 2), in the inner-lake water locations, points CH1 and CH2 (see Figure 2), compared with in-situ spectral data collected at the time of satellite overflight. As it can be observed, the spectral signature estimated by the atmospheric correction algorithms approximately matches with the data from the in-situ measurements. The best performance is appreciated in the visible wavelengths. Regarding the spectral reflectivity signatures in the inner-lake surface, as shown qualitatively in Figure 8 and quantitatively in Table 5, 6S and ATCOR cause a general overestimate in all wavelengths, while FLAASH is closer to the in-situ data and, consequently, achieves better statistical parameters (RMS and BIAS).

In general, the results show that correction applying algorithms based on the physical modelling are precise. Furthermore, these strategies obtain good estimations with low RMSE values. In summary, the superior performance of 6S model-based algorithm has been demonstrated providing the best overall accuracy for pre-processing WV-2 coastal shallow water imagery compared to ATCOR and FLAASH techniques, respectively. 

### 3.2. Coastal Monitoring: Benthos Abundance 

In this section, the results of coastal monitoring algorithms for mapping in shallow-water marine protected environments, detailed in Section 2.3, are presented, allowing the robust mapping of the spatial distribution and density of seagrass in coastal waters and, as mentioned in [11]: “Monitoring benthic habitats provides an important insight into the health of marine ecosystems, in general and in particular, algae-seagrass is a keystone of coastal, providing critical habitats and nutrients to fisheries.”

The algorithms performance have been evaluated in Corralejo-Isla de Lobos protected coastal ecosystem (Fuerteventura Island) where, in addition to being a UNESCO Biosphere Reserve, the monitoring of water quality is of special interest for its richness, marine life and aquatic touristic activities. In particular, an atmospherically corrected WV-2 multispectral image from Corralejo-Lobo channel, as shown in Figure 1c, without glint over the sea surface and clearly showing different types of seafloor, has been processed to highlight the benefits of our proposed methods to generate high-resolution satellite coastal value-added products. 

**Figure 9:**
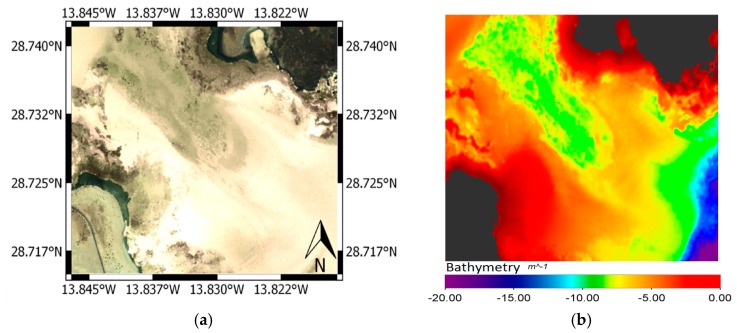
Results of model unmixing: (**a**) seafloor albedo (CB-G-B bands); (**b**) map of estimated depth (bathymetry).

Using the WV-2 multispectral bands, after the 6S atmospheric correction and the derived seafloor reflectivity, Figure 9a shows the RGB composite of the albedo computed by the linear mixing of the three pure classes and their abundances. High reflectivity of sandy covers can be appreciated. In turn, rocks leave the coast and extend below the sea, with very similar reflectivity. These rocks are modeled using the sediment class which has lower reflectivity than the other classes. Finally, areas with certain greenish hues can be observed, in the center of the channel and in the lower right side of the image, associated with the presence of small populations of algae and seagrass on the bottom. Figure 9b shows the results obtained in the radiative transfer modeling for the calculation of bathymetry. It can be observed the high level of resolution that satellite data, whose the degree of accuracy was studied in a previous work [11]. 

**Figure 10:**
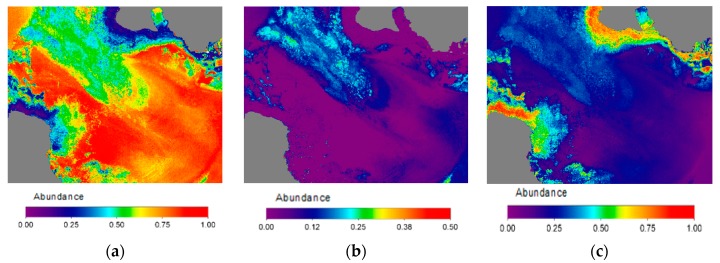
Result of the bottom lineal unmixing: (**a**) sand endmember abundance; (**b**) algae endmember abundance; (**c**) sediment-rock endmember abundance.

Figure 10a shows the abundance map of the sandy areas, dominant class in this coastal bottom. In the rocky abundance map, sand completely disappears in rocky areas and in zones where algae are located there is a mixture with the coastal bed reflectivity, which taking into account that depth of these zones is about 15 m makes it congruent the presence of algae or seagrass. The abundance of vegetation is shown in Figure 10b, with low abundance of about 20% in the center of the channel, suggesting a low algae or seagrass density. In the lower right of the map, an area with higher density, around 35%, corresponds to seagrass populations. Figure 10c shows the abundance of sediments, in this case rocks, which are close to the shore.

**Figure 11:**
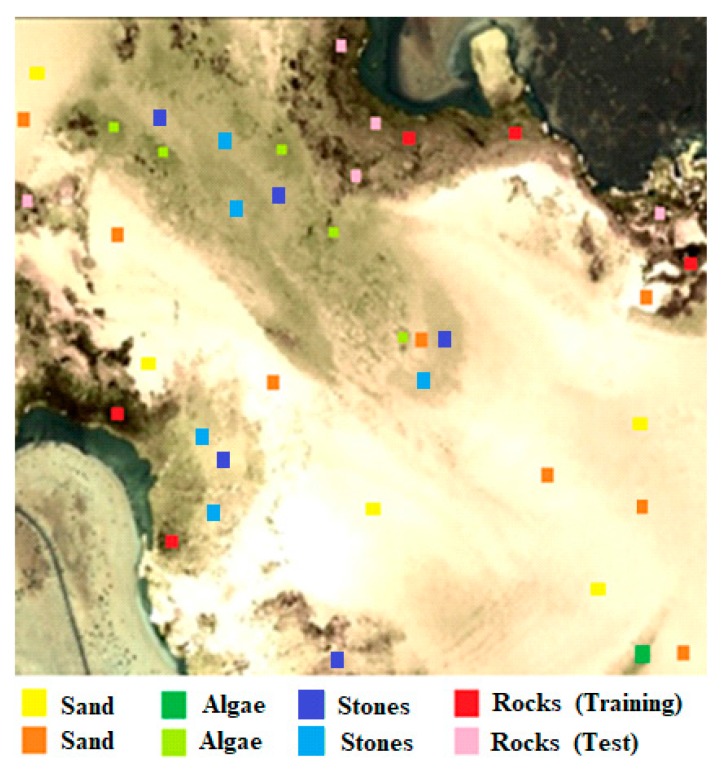
Training and test region for the classifier.

For the benthic classification, the three abundances of the modeled benthic classes plus the bathymetry have been introduced to the SVM classifier. The appropriate SVM parameters used were the radial basis function kernel, a gamma value of 0.25 and a penalty parameter of 100. To validate the classification result, as not a reliable seafloor map is available for the zone, data from expert marine biologists has been used. As 4 classes were considered by them, we have used the same four classes: sand, algae/seagrass, rock outcrops and a fourth class that includes stones-blocks-encrustations. Using that information, train and test regions have been selected, as shown in Figure 11.

Bathymetry provides some information about the stratification of some of the classes such as algae or seagrass, stones-encrustations and rock outcrop, for this reason it is used in the classification. A separability study of the different types of benthic classes was performed using the Jeffries-Matusita index. The computed separability between classes was very high, achieving values over 1.9, thanks to the fact that we work with the result of the linear unmixing algorithm, being fully constrained parameters.

**Figure 12:**
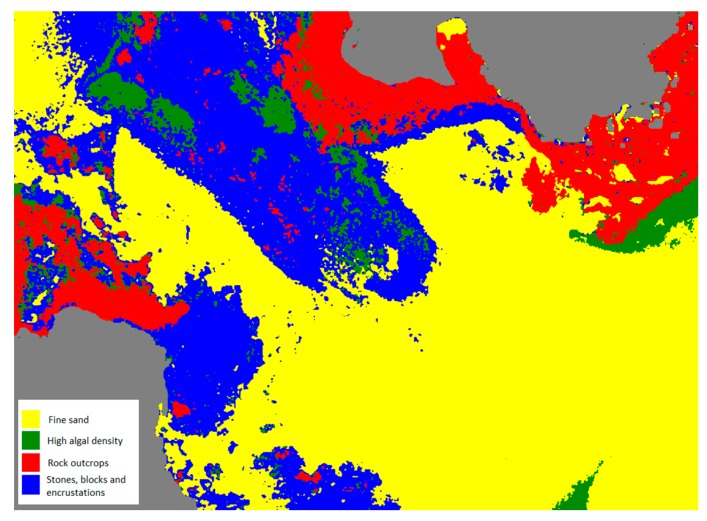
Result of the benthic high-resolution classification map using SVM and endmember abundances and bathymetry generated in the Marine Optical Model.

Figure 12 shows the WV-2 high-resolution benthic habitat composite map of Corralejo area, using the SVM classifier with the abundance and bathymetry information generated in the unmixing algorithm. After the classification, the confusion matrix using the test regions is shown in Table 5, providing an overall accuracy greater than 90% for the pure pixels selected as a reference. The biggest errors are obtained between algae and sand, because these classes are mixed, so the difficulty lies in defining what is the minimum value of vegetation necessary for the pixel to be classified as high density algae. Areas with high abundance in algae have been selected in the test and training regions. A small percentage of error can also be observed between the classification of the rock outcrop and the stones-blocks-encrustations, because their nature is similar.

## 4. Conclusions

An adequate and efficient monitoring of water quality parameters, bathymetry and distribution of benthic habitats of coastal waters ecosystems is important for life quality, global climate change and to guide decision-makers in governmental agencies, for example, in environmental protection, touristic activities, fisheries, etc. Therefore, coastal monitoring and the measurement of multi-temporal changes is an important tool in understanding our environment. 

High-resolution satellite-based imaging systems with spectral bands within the visible spectrum reliably provide information to implement spatially-based conservation actions and they enable observations of coastal parameters at broader spatial and finer temporal scales than those allowed through field observation alone.

Limitations in calibration, seasonal solar illumination geometry, viewing effects, atmospheric and sunglint disturbances have a certain impact on the results in coastal waters applications. As the water reflectivity is very weak in the high-spatial-resolution WorldView-2 data preprocessing strategy, adequate atmospheric correction and deglinting methods should be applied in order to increase the accuracy of the final products.

In this work, coastal locations at Canary Islands (Spain), Maspalomas and Corralejo Marine protected littoral zones, have been selected. Complex model-based atmospheric correction, FLAASH, ATCOR and 6S algorithms were implemented and statistically assessed comparing them with respect to in-situ measurement. Specifically, this work performed a comparison between the corrected spectral reflectivity of these three atmospheric correction algorithms, in Maspalomas coastal shallow waters and the inner-lake, with the reference signature measured with a spectroradiometer, acquired at the time of satellite overflight. 

It was demonstrated that model-based algorithms properly correct the atmospheric disturbances. Specifically, 6S achieved the best performance and, in particular, reached the lowest value of the RMSE for coastal shallow-water environments. On the other hand, FLAASH atmospheric correction algorithm worked properly in the inner-lake, where RMSEs are greater and all the bands are more affected.

The use of the 6S atmospheric model has not only provided a high accuracy atmospheric correction, as validated by in-situ data, but it has also given us insight about some useful information for the previously developed sunglint correction methodology based on physical principles. In this work, combined atmospheric correction and an automatic sunglint removal algorithm, which would allow us to eliminate the contribution of the specular NIR reflectance, has been applied to the successful generation of bathymetry and distribution of benthic habitats in the shallow-water environments.

The mapping of benthic habitats is a complex problem because only limited and noisy spectral information is available. In this context, for the application of pre-processed WorldView-2 multispectral imagery to estimate the benthic habitat mapping and to retrieve bathymetry information, an efficient multichannel physics-based model has been implemented. The sophisticated model developed and evaluated in this study expands the previous developed methodology which was based on combination of water column correction, seafloor types normalized indexes and supervised classification techniques. The enhanced capacity provided by the WorldView-2 imagery, coupled with the new model presented herein, providing an albedo estimation of the coastal bottom by linear unmixing of the pure benthic elements and their abundances, obtains better precision in benthic habitat estimation.

In summary, WorldView-2 processing methodology has provided a systematic and a synoptic framework for improving the scientific knowledge about littoral zones and to properly retrieve coastal shallow water parameters. This approach has been validated over a database of in-situ measurements collected during field campaigns. The excellent results provided by these studies have been successfully applied to the generation of benthic habitat and bathymetry maps of natural protected ecosystems in Canary Islands.

## Figures and Tables

**Table 1:** Review of recent atmospheric correction studies categorized as image-based and physical model-based.

Category	Algorithm	Authors	HR Satellite-based System
Image-based	Dark Object Subtraction (DOS)	Wu et al. (2005) [15]	QuickBird Landsat ETM+ WorldView-2
		Nguyen et al. (2015) [16]	
		Martin et al. (2012) [23]	
	Cosine of the sun zenith angle (COST)	Wu et al. (2005) [15]	QuickBird Geoeye and Rapideye WorldView-2
		Broszeit and Ashraf (2013) [17]	
		Martin et al. (2012) [23]	
	QUick Atmospheric Correction (QUAC)	Agrawal and Sarup (2011) [18]	Hyperion QuickBird and WorldView
		Pacifici (2013) [24]	
Physical model-based	Fast Line-of-sight Atmospheric Analysis of Spectral Hypercubes (FLAASH)	Nguyen et al. (2015) [16]	Landsat ETM+ Hyperion WorldView-2 QuickBird and WorldView Hyperion
		Agrawal and Sarup (2011) [18]	
		Pu et al. (2015) [19]	
		Pacifici (2013) [24]	
		San et al. (2010) [25]	
	ATmospheric CORrection (ATCOR)	Broszeit and Ashraf (2013) [17]	Geoeye and Rapideye Landsat TM and ETM+
		Vanonckelen et al. (2015) [20]	
	Second Simulation of a Satellite Signal in the Solar Spectrum (6S)	Nguyen et al. (2015) [16]	Medium Resolution Spot 5 WorldView-2
		El Hajj et al. (2008) [21]	
		Martin et al. (2012) [23]	

**Table 2:** Information about WorldView-2 imagery and validation instrumentation used in this work.

Area	Date/Time	Latitude (°N)	Longitude (°W)	Field Data Acquisition
Maspalomas	11 August 2013	UL: 27.7785459	UL: 15.6810007	Reflectance (ADS Fieldspec 3), water quality parameters, bathymetry (Reson Navisound 110 echosounder) and GPS location (trimble DSM132).
	12:05:24 UTC	LR: 27.7139141	LR: 15.5318325	
Maspalomas	4 June 2015	UL: 27.7784654	UL: 15.6971379	Reflectance (ADS Fieldspec 3), water quality parameters, bathymetry (Reson Navisound 110 echosounder), seafloor video (GoPro Hero 3+) and GPS location (trimble DSM132).
	11:49:47 UTC	LR: 27.7099647	LR: 15.5306367	
Corralejo Lobos	28 October 2010	UL: 28.7443727	UL: 13.8561800	No field data measurements
	11:51:00 UTC	LR: 28.6306247	LR: 13.8110141	

(UL: Upper Left, LR: Lower Right).

**Table 3:** Location of corresponding in-situ sampling sites of Figure 2.

Point	Latitude	Longitude
A1	27°45′03.49″	15°33′51.05″
A3	27°45′03.17″	15°33′40.18″
C1	27°43′58.80″	15°35′13.09″
C3	27°43′27.01″	15°35′14.93″
D1	27°43′57.72″	15°36′05.08″
D3	27°43′41.84″	15°36′07.63″
E1	27°44′20.04″	15°36′31.00″
E3	27°44′04.34″	15°36′48.53″
F1	27°44′46.50″	15°37′08.72″
F3	27°44′14.21″	15°37′06.67″
G1	27°44′41.86″	15°37′33.35″
G3	27°44′18.17″	15°37′33.06″
CH-1	27°44′12.41″	15°35′38.57″
CH-2	27°44′15.28″	15°35′37.05″

**Table 4:** RMSE and BIAS between the in-situ measurements and satellite corrected reflectance for each atmospheric algorithm (best results in bold).

Algorithm	Scenario	RMSE	BIAS
**FLAASH**	Coast	0.0379	−0.0355
	Inner-lake	**0.0141**	**−0.0034**
**ATCOR**	Coast	0.0318	−0.0251
	Inner-lake	0.0185	0.0143
**6S**	Coast	**0.0271**	**−0.0217**
	Inner-lake	0.0153	0.0089

**Table 5:** Confusion Matrix using ground truth.

	Ground Truth (Percent)					
	Overall Accuracy = 93.32%. Kappa Coefficient = 0.87					
	Stones	Rock	Sand	Algae	% of Total	Accuracy
**Stone**	93.56	2.58	0.09	1.72	19.03	93.56
**Rock**	5.76	96.58	0.00	0.96	23.80	96.58
**Sand**	0.69	0.82	92.31	2.39	50.88	92.31
**Algae**	0.00	0.02	7.60	94.93	6.29	94.93
